# Cellular Factors Targeting HIV-1 Transcription and Viral RNA Transcripts

**DOI:** 10.3390/v12050495

**Published:** 2020-04-29

**Authors:** Rayhane Nchioua, Matteo Bosso, Dorota Kmiec, Frank Kirchhoff

**Affiliations:** 1 Institute of Molecular Virology, Ulm University Medical Center, 89081 Ulm, Germany; nchioua.rayhane@uni-ulm.de (R.N.); matteo.bosso@uni-ulm.de (M.B.); 2 Department of Infectious Diseases, King’s College London, Guy’s Hospital, London SE1 9RT, UK; dorota.kmiec@kcl.ac.uk

**Keywords:** HIV, restriction factors, ZAP/KHNYN, N4BP1, Sp1, RNAses, viral latency

## Abstract

Restriction factors are structurally and functionally diverse cellular proteins that constitute a first line of defense against viral pathogens. Exceptions exist, but typically these proteins are upregulated by interferons (IFNs), target viral components, and are rapidly evolving due to the continuous virus–host arms race. Restriction factors may target HIV replication at essentially each step of the retroviral replication cycle, and the suppression of viral transcription and the degradation of viral RNA transcripts are emerging as major innate immune defense mechanisms. Recent data show that some antiviral factors, such as the tripartite motif-containing protein 22 (TRIM22) and the γ-IFN-inducible protein 16 (IFI16), do not target HIV-1 itself but limit the availability of the cellular transcription factor specificity protein 1 (Sp1), which is critical for effective viral gene expression. In addition, several RNA-interacting cellular factors including RNAse L, the NEDD4-binding protein 1 (N4BP1), and the zinc finger antiviral protein (ZAP) have been identified as important immune effectors against HIV-1 that may be involved in the maintenance of the latent viral reservoirs, representing the major obstacle against viral elimination and cure. Here, we review recent findings on specific cellular antiviral factors targeting HIV-1 transcription or viral RNA transcripts and discuss their potential role in viral latency.

## 1. Introduction

Since the beginning of the pandemic, about 80 million people have been infected with HIV-1 and approximately half of them have died of AIDS. It is estimated that, in total, about 1.7 million individuals became newly infected and about 770,000 people died from AIDS-related illnesses in 2018 (NIH global AIDS report). Although these numbers are still sobering, they illustrate that significant progress has been made in the fight against AIDS, because the availability of combined antiretroviral therapy (cART) has allowed for the reduction of AIDS-related deaths by more than half since the peak in 2004. In 2018, approximately 62% of around 37.6 million people who knew their infection status had access to cART. Infected individuals on cART can have an almost normal life expectancy with essentially no risk of transmitting the virus to uninfected individuals, since effective treatment leads to undetectable viremia. However, cART usually must be taken daily and for life since it does not cure the HIV infection. To date, only two individuals, known as the Berlin and London patients, have been reported to be cured of HIV-1 infection, both following CCR5Δ32/Δ32 haematopoietic stem-cell transplantation—a method that is risky and unfortunately not broadly applicable [1,2].

The key hurdle to the elimination of HIV from the human body is the ability of this virus to establish highly stable latent reservoirs in long-living cells. Reverse transcription of the single-stranded HIV-1 RNA into linear double-stranded DNA, and the subsequent integration of the viral genome into that of the host cell, are essential steps of the retroviral replication cycle. In most cases, HIV-1-infected CD4^+^ T cells will initiate efficient proviral transcription and the production of progeny HIV virions. While these productively infected cells account for the bulk of viremia in untreated HIV-infected individuals, they are rapidly eliminated after the initiation of effective antiretroviral therapy (ART). In some cases, however, HIV-1 integrates into the human chromosomal DNA, but the provirus remains transcriptionally silent. In this form, the latently infected cell is not recognized and eliminated by the immune system or targeted by ART. Thus, persistence of HIV in long-living memory CD4^+^ T cells prevents full viral clearance, even after decades of effective treatment, and represents the main obstacle to a cure of HIV/AIDS [3,4]. One approach to target these reservoirs that is intensively pursued is the so called “shock/ kick and kill” approach [5,6]. It involves two major steps, i.e., treatment with a combination of latency-reversing agents to reactivate latent HIV hiding in the immune cells (the “shock/kick”), and targeting them for elimination by immune mechanisms (the “kill”) while preventing new infections by cART. Unfortunately, it has proven highly challenging to achieve potent reactivation of the highly heterogenous proviral reservoirs and to achieve efficient elimination of the virus-producing cells [7]. Thus, an alternative opposite strategy, called “block and lock” is also considered. It aims to permanently silence the transcription of all proviruses, even after treatment interruption [5,8]. Block and lock approaches to achieve a cure of HIV/AIDS are still at an early stage and unlikely to permanently eliminate or inactivate all replication-competent HIV. Thus, HIV might re-emerge and continuous drug treatment, similar to current ART, is required [9].

The establishment and maintenance of HIV-1 latency is determined by numerous mechanisms. These involve the availability of cellular transcription and elongation factors, epigenetic modifications, the site of proviral integration, as well as effectiveness of RNA splicing, nuclear export, translation and immune clearance of virally infected cells [3,4,10,11,12,13,14]. In addition, certain properties of the accessory genes of HIV-1, which modulate the state of activation of the infected cells and may prevent immune clearance, also play a role [15]. Some of the major mechanisms governing HIV latency have been extensively studied and have been the topic of excellent recent reviews [7,11,16,17,18,19]. Here, we focus on cellular factors suppressing HIV proviral transcription by targeting the host transcription factor specificity protein 1 (Sp1). In addition, we summarize recent progress in the characterization of cellular RNA interacting factors capable of degrading viral RNA transcripts after proviral integration. We hope that the present review will encourage further studies on the potential role of cellular factors modulating viral transcription and targeting viral RNA transcripts in HIV latency.

## 2. Targeting Sp1 for Transcriptional Silencing

**Figure 1:**
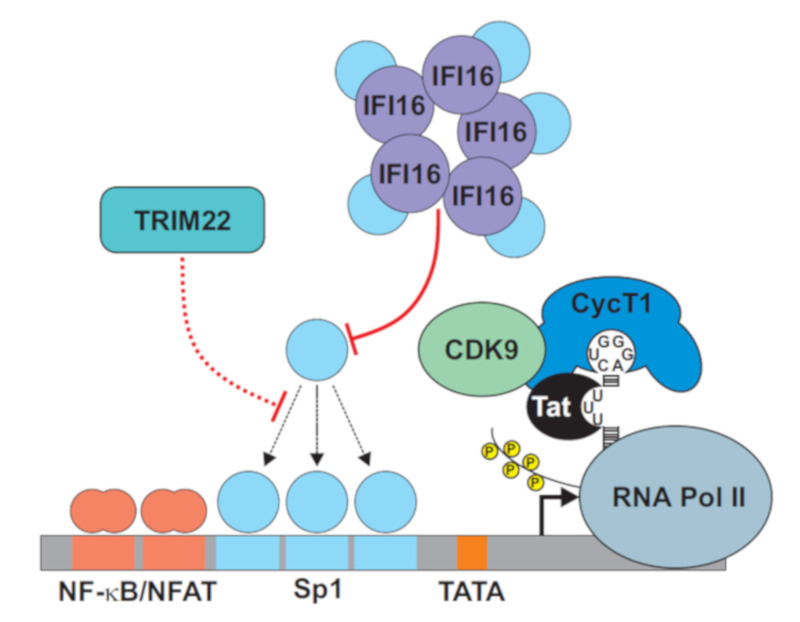
Specificity protein 1 (Sp1) targeting suppresses HIV-1 transcription. As outlined in the text, the viral transactivator of transcription protein (Tat) interacts with the TAR region at the 5’ end of the viral RNA and recruits several cofactors for effective viral transcription. The latter is also dependent on the availability of the cellular transcription factors nuclear factor kappa B (NF-κB) and Sp1. Cellular factors tripartite motif-containing protein 22 (TRIM22) and γ-IFN-inducible protein 16 (IFI16) target Sp1 to suppress HIV-1 transcription and reactivation from latency.

The transcription of HIV-1 depends on a complex interplay between numerous viral and cellular factors (Figure 1). Key players of productive HIV-1 infection, such as the viral transactivator of transcription (Tat) and the regulator of virion expression (Rev), as well as cellular cofactors, e.g., the positive transcription elongation factor complex (P-TEFb) and RNA polymerase II (RNAPII), have been well studied [17,18,20,21]. In addition to changes in these factors, latent HIV-1 infection can be promoted by transcriptional repressors that may recruit histone deacetylases (HDAC) to the LTR promoter [22,23], or by the absence of host elongation and transcription factors [24,25].

It has been established that host transcriptional regulators such as nuclear factor kappa B (NF-κB), nuclear factor of activated T cells (NFAT), activating protein-1 (AP-1), and specificity protein 1 (Sp1) are important for HIV-1 transcription and latency. NF-κB strongly enhances HIV-1 transcription and can be activated by extracellular stimuli, such as T-cell receptor (TCR) ligands and various cytokines. Thus, NF-κB is a major target in HIV-1 cure research, and its stimulation to activate latent HIV-1 has been intensively studied [17,26,27,28]. In support of the important role of this transcription factor, recent studies revealed that HIV-1 has evolved sophisticated mechanisms to fine-tune NF-κB activity throughout the replication cycle to support viral transcription while minimizing antiviral gene expression [29,30,31]. NFAT recognition sequences on the viral promoter overlap with NF-κB target elements, and the binding of either one is mutually exclusive [32]. Thus, it has been proposed that NF-κB and NFAT might act in a sequential manner, with NF-κB being critical for the initial response and NFAT acting at later time points [33]. It has been reported that the induction of NFAT activity by IL-7 stimulates naïve T cells and consequently affects HIV-1 infection and latency [34]. While NF-κB activators are capable of reactivating latent HIV-1, they also bear significant risks of adverse effects, since NF-κB regulates numerous physiological processes [35].

HIV-1 LTRs usually contain three tandem Sp1 binding sites, and early studies established that Sp1 plays an important role in regulating viral transcription [36,37,38]. Compared to NF-κB, however, the role of Sp1 in HIV-1 latency has received little attention. One reason for this is that Sp1 is commonly thought to be ubiquitously expressed and not limiting to proviral transcription. However, recent evidence shows that several interferon (IFN)-inducible antiviral factors may target Sp1 to suppress HIV transcription and play roles in viral latency (Figure 1). Initially, it has been reported that the tripartite motif-containing protein 22 (TRIM22), which shows broad antiviral activity [39] and is strongly up-regulated by type I IFN, suppresses HIV-1 transcription by the inhibition of Sp1 binding to the viral promoter [40]. This antiviral activity of TRIM22 was independent of its E3 ubiquitin ligase activity, Tat, and NF-κB and not associated with alterations in the total cellular levels of Sp1 [41,42,43,44]. More recently, the group provided further evidence that TRIM22 suppresses the reactivation of latent HIV-1, at least in CD4^+^ T-cell lines, and this effect was dependent on Sp1 binding sites in the viral LTR promoter [45]. However, the mechanism remains obscure, since TRIM22 does not directly interact with Sp1 nor with the viral promoter. The indirect effects of TRIM22 on Sp1 binding to the HIV-1 LTR, could involve the activation of cellular factors promoting a transcriptionally silenced heterochromatin configuration, the induction of post-translational modifications of Sp1, or the increased binding of Sp3, another Sp family member that might repress transcription [40,46].

More recently, it has been shown that γ-IFN-inducible protein 16 (IFI16) restricts HIV-1 by sequestering the transcription factor Sp1 and inhibiting viral gene expression [47]. Thus, IFI16 might play a complex role in HIV-1 infection and latency, since it has previously been characterized as a cytosolic immune sensor of HIV-1 DNA species that boosts IFN induction in macrophages [48,49]. In addition, it has been reported that IFI16 senses reverse transcription (RT) intermediates in tissue CD4^+^ T cells that are abortively infected with HIV-1, resulting in highly inflammatory caspase-1 dependent pyroptotic cell death [50]. However, the main function of IFI16 as a cytosolic sensor of viral DNA species was at odds with the predominantly nuclear localization of this factor. Indeed, recent studies showed that IFI16 suppresses the transcription of various DNA viruses in the nucleus independently of innate immune sensing [51,52,53,54,55,56]. A variety of non-exclusive mechanisms underlying the antiviral activity of IFI16 have been suggested, including global histone modifications, alterations in viral chromatin structures, as well as the binding to and occupation of viral promoters. The most elegant model proposes that IFI16 cooperatively binds dsDNA via its HIN domains in a length-dependent manner and interactions between the pyrin domain mediate the assembly into protein filaments [57]. It has been suggested that IFI16 might initially interact with G-quadruplex (G4) structures in DNA [58], which frequently also arise within Sp1 binding sites [59], and that the subsequent filament formation mediates the high affinity binding of DNA [57]. Since foreign DNA entering the nucleus is better accessible than self-DNA, this mechanism would explain how IFI16 distinguishes self from non-self, as well as why it interferes with the activity of Sp1-dependent promoters. In agreement with such a mechanism, it has been reported that IFI16 interacts and colocalizes with the genomes of various herpesviruses within the nucleus [51,52,53,60,61]. However, the HIN domains, and hence the DNA binding, were fully dispensable for the ability of IFI16 to suppress HIV-1 transcription. Instead, the pyrin domain of IFI16 competed with the DNA for Sp1 binding, resulting in reduced availability of this transcription factor for proviral transcription. Notably, Sp1 inhibitors have been approved for clinical use in cancer therapy [62], and Mithramycin A efficiently suppressed HIV-1 reactivation in primary CD4+ T cells [47]. Thus, Sp1 might represent a suitable target for “block and lock” approaches [8,16].

The finding that at least two IFN-inducible factors target Sp1 to restrict HIV-1 suggests that this transcription factor may become limiting, especially during acute infection when IFN levels are high and the latent viral reservoirs are established. In agreement with this possibility, Sp1 frequently seems to be a limiting factor for efficient HIV-1 transcription in T cells [47]. It has long been known that the LTR promoter of the most prevalent HIV-1 subtype C shows some differences from other less common subtypes of HIV-1, such as an additional NF-κB binding site [63]. Recent evidence suggests that subtype C HIV-1 strains are less dependent on Sp1 for effective transcription and less sensitive to inhibition by IFI16, which seems to suppress viral reactivation from latency [47]. Altogether, further studies on the role of Sp1 and its IFN-inducible inhibitors, i.e., TRIM22 and IFI16, as well as subtype-specific differences in the establishment and maintenance of latent HIV-1 infections, seem highly warranted.

## 3. Cellular Factors Targeting HIV-1 RNA Transcripts

**Figure 2:**
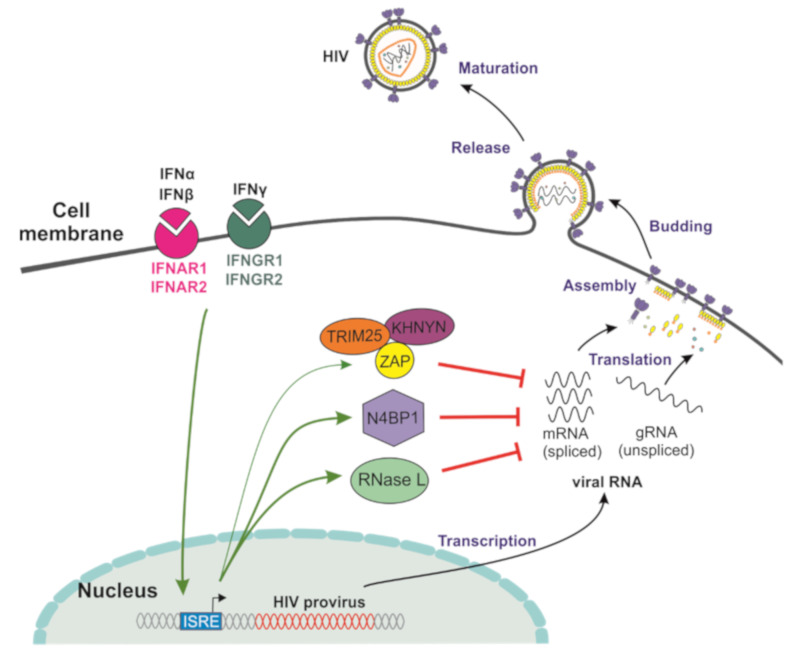
Factors targeting HIV-1 RNA transcripts. Schematic presentation of cellular factors targeting viral RNAs to suppress translation of HIV-1 proteins and/or packaging of genomic viral RNAs.

Restriction factors may target viral pathogens at every step of their replication cycle and a large variety of cellular factors target viral transcripts for degradation [64,65,66] (Figure 2). Degradation of viral RNAs, and, consequently, decreased viral protein expression, might prevent the elimination of infected cells by the immune system and thus promote the establishment of latent viral reservoirs. In addition, the effective degradation of viral transcripts might prevent HIV-1 from entering the productive cycle and allow HIV-1 to maintain a latent state. One important effector of innate antiviral immunity that activates the NLRP3 inflammasome during viral infections is RNase L [67,68]. The monomeric form of this enzyme is inactive but dimerizes and becomes active upon the binding of 2’-5’oligoadenylate (2’-5’A) that is generated by 2’-5’ oligoadenylate synthetase (OAS) transcription and modification. It has been reported that viral RNA cleavage by RNAse L is important for an effective and sustained antiviral IFN response induced by viral dsRNA [69]. Notably, only OAS3, not OAS1 and 2, is critical for the activation of RNase L during infection by diverse RNA and DNA viruses [70]. Upon activation, RNase L is broadly active and destroys viral as well as cellular RNAs [71]. Interestingly, HIV-1 may induce the expression of the RNase L inhibitor to attenuate inhibition by the 2-5A/RNase L pathway [72]. The degradation of all RNAs within a cell usually occurs together with a shut-down of translation activity induced by protein kinase R (PKR) and represents the cell’s last effort to defeat a virus before triggering apoptosis [73,74]. Consequently, RNA degradation might lead to the activation of melanoma differentiation-associated protein 5 (MDA5), an RNA helicase involved in the production of IFN-β [75]. Notably, RNase L is not only involved in antiviral innate immunity. It has been reported that RNase L protects the central nervous system against demyelination during viral infection [76] and plays a key role in senescence [77]. In addition, RNase L might function as a tumor suppressor, and defects in the OAS/RNase L pathway have been detected in prostate cancer and chronic fatigue syndrome [78,79]. It is conceivable that factors affecting HIV-1 transcription and cell survival might play a role in viral latency. However, the potential role of the OAS/RNase L system in the establishment and maintenance of the latent viral reservoirs remains to be examined.

Another factor targeting viral RNAs is the zinc finger antiviral protein (ZAP). This protein exerts a broad antiviral activity and does not only restrict retroviruses, such as HIV-1 and MLV, but also numerous other RNA and DNA viruses (reviewed in Ghimire et al., 2018 [80]). In addition, ZAP targets retroelements as well as some cellular RNAs. Interestingly, it has been reported that ZAP requires RNase L and OAS3 for the effective restriction of Echovirus 7 [81]. ZAP, also called PARP13, belongs to the poly (ADP-ribose) polymerase (PARP) family, which has seventeen members in humans [82]. PARP proteins use NAD+ to transfer ADP-ribose to various target proteins to post-translationally regulate their stability and function. PARP proteins are ubiquitously expressed in many cell types and are involved in numerous processes including cell division and survival, the regulation of chromatin structure, and DNA damage repair [82,83,84,85]. It has long been known that ZAP exerts antiviral activity [86], but the mechanism by which it can recognize foreign elements remained unclear. Many RNA viruses mimic the CpG suppression of their vertebrate hosts [87,88] and increasing the abundance of this dinucleotide in the genomes of HIV, influenza A virus, or picornaviruses is detrimental to their replication [89,90,91]. Bieniasz and colleagues showed that ZAP is poorly active against wild-type HIV-1 but efficiently restricts the virus variants with artificially enriched CpG content [92]. They further confirmed that ZAP selectively binds to CpG-rich RNA sequences and proposed that it exploits host CpG suppression to recognize and deplete foreign RNAs. The crystal structure of the N-terminal RNA-binding domain of human ZAP and a CpG dinucleotide-containing RNA showed that a cavity on the ZAP surface can accommodate a CpG dinucleotide but no other dinucleotides, which explains the specificity [93].

ZAP is expressed in several isoforms [94] and is catalytically inactive, probably due to the lack of a crucial “His-Tyr-Glu” motif in its PARP domain. It has been reported that residues in the PARP-like domain found in the longer isoforms of ZAP (ZAP-L) evolved under positive selection during primate evolution and might be required for full activity against alphaviruses and retroviruses [83]. ZAP-L has been reported to represent the most antivirally active isoform but is not significantly upregulated by IFN, while the shorter ZAP-S also exerts antiviral activity and is IFN-inducible but may actually act as a negative feedback regulator of the interferon response [95]. Depletion of ZAP reduced the inhibitory effect of type I IFN on CpG-enriched HIV-1, suggesting that this factor contributes to the antiviral IFN response [96]. Importantly, ZAP needs cofactors to exert antiviral activity. Initial findings showed that the tripartite motif-containing protein 25 (TRIM25) mediates K48 and K63-linked poly-ubiquitination of ZAP and proposed that TRIM25 is required for the optimal binding of ZAP to target mRNA [97,98]. However, the ubiquitination of ZAP is not required for its antiviral activity, and recently it has been shown that ZAP also requires KHNYN to restrict CpG-enriched HIV-1 [99]. Unlike ZAP and TRIM25, KHNYN contains an endonuclease domain and thus most likely not only has the capacity to interact with RNA but also to destroy it. KHNYN interacts with ZAP as well as TRIM25 and requires both to selectively inhibit HIV-1 with clustered CpG dinucleotides. The exact roles of the three proteins in HIV-1 restriction need further investigation. Notably, the inhibitory effect of ZAP on HIV-1 and other primate lentiviruses is not only determined by CpG frequency. Using different approaches, i.e., artificial CpG-enrichment in specific regions of the HIV-1 genome [96] and analyses of a large panel of primary infectious molecular clones of HIV-1 [100], two recent studies showed that the CpG frequency in first part of the viral *env* gene, rather than the overall content, determines ZAP sensitivity. The latter study also showed that the genomes of different primate lentiviruses differ substantially in CpG frequencies, and that the magnitude of suppression does not correlate with ZAP sensitivity, suggesting possible viral evasion or counteraction mechanisms.

The role of ZAP and its cofactors in HIV-1 latency remains to be determined. On the one hand, ZAP-driven CpG suppression might promote productive infection, since it reduces sites for CpG methylation [101] that might induce transcriptional silencing of the HIV-1 LTR promoter [102]. On the other hand, elimination of viral RNA and decreased antigen expression might reduce the elimination of virally infected T cells, allowing them to return to a resting phenotype and become latent viral reservoirs. It has been reported that ZAP might play a role in regulating herpesvirus latency [103], and the knock-down of endogenous ZAP moderately enhanced the expression of Human T-cell leukaemia virus type 1 (HTLV-1) mRNA and proteins [104]. Despite significant CpG suppression, primary HIV-1 strains are not fully resistant against ZAP inhibition, and correlative analyses indicate that CpGs in the *env* region governing ZAP sensitivity might affect viral replication and disease progression *in vivo* [100]. Further studies on the role of cellular factors targeting HIV-1 RNA transcripts in the establishment and maintenance of latent infection seem highly warranted.

Just recently, NEDD4-binding protein 1 (N4BP1) has been identified as a potent HIV-1 restriction factor [105]. Notably, N4BP1 shares CGIN1 and NYN domains with KHNYN, described above [106]. N4BP1 is strongly inducible by type I IFN in primary T cells and suppresses HIV-1 replication by binding and degrading viral mRNA. Importantly, N4BP1 is cleaved and consequently inactivated by MALT1, a protease that is induced in activated CD4^+^ T cells [105]. MALT1-mediated cleavage of N4BP1 promoted reactivation of latent HIV-1 proviruses during T-cell activation. Thus, N4BP-1 controls HIV-1 latency and reactivation at a post-transcriptional level, and its inactivation by MALT1 might represent a useful target in the “kick” part of cure strategies. Notably, MALT1 targets a variety of additional RNases (e.g., Regnase-1, Roquin-1 and Roquin-2) controlling lymphocyte activation by regulating RNA stability. For Regnase-1, which is also referred to as monocyte chemotactic protein-induced protein 1 (MCPIP1), the restriction of HIV-1 in unstimulated CD4+ T cells has already been demonstrated [107]. Thus, further studies on the antiretroviral activity of these cellular RNAses are highly warranted. In addition, it will be of significant interest to determine whether the MALT1-dependent cleavage of N4BP1, Regnase-1, and other RNases plays a major role in viral reactivation from latency and hence the rebound of HIV-1 after treatment interruption.

## 4. Summary and Perspectives

Viral latency has become a major research focus since it represents the main hurdle against a cure of HIV/AIDS. It has been established that HIV-1 latency can be determined by numerous mechanisms, including those involving the site of proviral integration, viral accessory and regulatory gene functions, the availability of cellular transcription and elongation factors, epigenetic modifications, viral RNA splicing, nuclear export, stability and translation, as well as immune clearance and survival times of virally infected cells. Nonetheless, we are still far from a full understanding of the mechanisms underlying the establishment and maintenance of the latent reservoirs of HIV-1. Inhibitors of Sp1 are already clinically approved and might be useful for block and lock approaches. In addition, inhibition or enhanced protease-mediated inactivation of cellular factors targeting viral RNAs may help to eliminate virally infected cells upon the reactivation of latent HIV-1 proviruses.
